# Variants in *HCFC1* and *MN1* genes causing intellectual disability in two Pakistani families

**DOI:** 10.1186/s12920-024-01943-2

**Published:** 2024-07-02

**Authors:** Syeda Iqra Hussain, Nazif Muhammad, Shahbaz Ali Shah, Adil u Rehman, Sher Alam Khan, Shamim Saleha, Yar Muhammad Khan, Noor Muhammad, Saadullah Khan, Naveed Wasif

**Affiliations:** 1 Department of Biotechnology and Genetic Engineering, Kohat University of Science & Technology (KUST), Kohat, Khyber Pakhtunkhwa Pakistan; 2 Department of Computer Science and Bioinformatics, Khushal Khan Khatak University, Karak, Pakistan; 3 Department of Biotechnology, University of Science and Technology, Bannu, Pakistan; 4 Institute of Human Genetics, Ulm University and Ulm University Medical Center, 89081 Ulm, Germany; 5 Institute of Human Genetics, University Hospital Schleswig-Holstein, Campus Kiel, Kiel, Germany

**Keywords:** Intellectual disability, Exome sequencing, *HCFC1* gene, *MN1* gene, Sanger Sequencing

## Abstract

**Background:**

Intellectual disability (ID) is a neurodevelopmental condition affecting around 2% of children and young adults worldwide, characterized by deficits in intellectual functioning and adaptive behavior. Genetic factors contribute to the development of ID phenotypes, including mutations and structural changes in chromosomes. Pathogenic variants in the *HCFC1* gene cause X-linked mental retardation syndrome, also known as Siderius type X-linked mental retardation. The *MN1* gene is necessary for palate development, and mutations in this gene result in a genetic condition called CEBALID syndrome.

**Methods:**

Exome sequencing was used to identify the disease-causing variants in two affected families, A and B, from various regions of Pakistan. Affected individuals in these two families presented ID, developmental delay, and behavioral abnormalities. The validation and co-segregation analysis of the filtered variant was carried out using Sanger sequencing.

**Results:**

In an X-linked family A, a novel hemizygous missense variant (c.5705G > A; p.Ser1902Asn) in the *HCFC1* gene (NM_005334.3) was identified, while in family B exome sequencing revealed a heterozygous nonsense variant (c.3680 G > A; p. Trp1227Ter) in exon-1 of the *MN1* gene (NM_032581.4). Sanger sequencing confirmed the segregation of these variants with ID in each family.

**Conclusions:**

The investigation of two Pakistani families revealed pathogenic genetic variants in the HCFC1 and MN1 genes, which cause ID and expand the mutational spectrum of these genes.

**Supplementary Information:**

The online version contains supplementary material available at 10.1186/s12920-024-01943-2.

## Introduction

Genetic aberrations in the human genome suppress the mental growth, leading to neurodevelopmental anomalies. Intellectual disability (ID) with speech delay and behavioral abnormalities ranges in severity from mild to profound, and can be associated with other conditions like epilepsy, sensory impairment, and autism spectrum disorders [1]. A definitive diagnosis is made based on a person’s IQ test score, calculated below 70 [2]. Several genes, including HCFC1 and MN1, have been reported in the literature, causing intellectual disability of various intensities.

The Host Cell Factor C1 (*HCFC1*) gene (OMIM# 300,019) synthesizes a protein called host cell factor C1 (HCFC1). This particular protein plays a crucial role in many cellular processes, encompassing gene transcription, DNA replication, and chromatin remodeling [3]. The mutations in the *HCFC1* gene have been observed to be correlated with the manifestation of genetic disorders, including an X-linked syndrome, known as methylmalonic aciduria and homocysteinemia, cblX type, characterized by cognitive impairments, delays in developmental milestones, and additional neurological indications like behavioral problems [4–6].

The meningioma-1 (*MN1*) gene (OMIM# 618,774) is a transcription activator that controls osteoblast proliferation, motility, differentiation, and function in mammals and is essential for the development of the mammalian palate [7]. C-terminal truncating variants of the MN1 transcriptional factor have been recently reported to cause a distinct phenotype characterized by craniofacial anomalies and partial rhombencephalon synapsis, a rare brain malformation characterized by midline fusion of the cerebellar hemispheres with partial or complete loss of the cerebellar vermis [8]. The manifestation of this condition, also known as MN1 C-terminal truncation (MCTT) syndrome or CEBALID (Craniofacial deformities, dysmorphic Ears, Brain Abnormalities, Language delay, and Intellectual Disability), results from the dominantly active truncated protein of MN1 rather than haploinsufficiency [9]. Intellectual disability, developmental delay, hypotonia (low muscle tone), and psychomotor retardation (delayed or impaired motor skills) are hallmarks of CEBALID syndrome. Cutis laxa, facial dysmorphism, and cerebellar abnormalities may also be associated with this syndrome [10].

The current study aims to investigate the genotype-phenotype correlation of affected individuals in two Pakistani families exhibiting various ID phenotypes. Exome sequencing revealed disease-causing variants in these families, increasing their mutational spectrum.

## Materials and methods

### Collection of blood samples

**Fig. 1:**
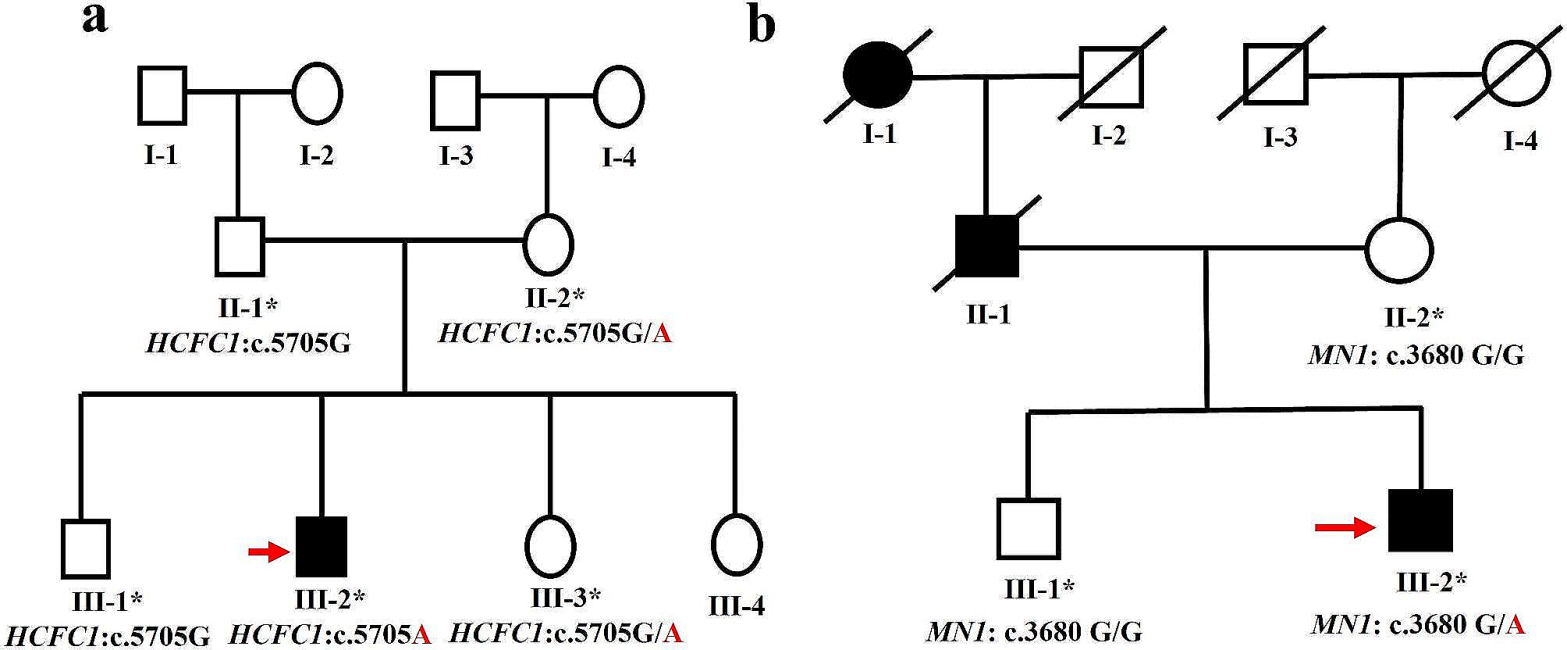
**(a)** Pedigree of family A showing X-linked pattern of ID and unaffected and affected individuals of the family **(b)** Pedigree of family B showing autosomal dominant pattern of ID and the unaffected and affected individuals of the family

The institutional ethical review board of Kohat University of Science and Technology, Kohat, Pakistan, approved the study (Ref No./KUST/Ethical committee/2191 dated. 06/10/2021), carefully following the recommendations of the Declaration of Helsinki. Two families (A and B) with ID were enrolled in the current study. Family A, from the Karak region of Khyber Pakhtunkhwa, Pakistan, consisted of three generations and presented an X-linked mode of inheritance. Five individuals, including four unaffected individuals (II-1, II-2, III-1, and III-3) and an affected individual (III-2), participated in this study. Family B, showing the autosomal dominant mode of inheritance, was recruited from Khairpur district of Sindh province. Three members participated in the study, including two unaffected individuals (II-2 and III-1) and one affected (III-2). Adults from both families were interviewed to compile the disease history and construct the pedigrees (Fig. 1A-B). The blood samples were collected into BD Vacutainer^®^ (BD, Franklin Lakes, NJ, USA) blood collection tubes and preserved after both families gained informed and written consent. Standard phenol-chloroform extraction methods were used to isolate DNA from the blood samples [11], and the concentration of the extracted DNA was measured using Qubit Fluorometer (Thermo Fisher Scientific Inc., Waltham, MA, USA) to an accuracy of 40 ng/µl.

### Whole exome sequencing

DNA samples (Family A-III-2; Family B-III-2) of two intellectually disabled patients belonging to two ethnically different families were used for whole exome sequencing, which was followed by data filtration to remove variances.

Using the xGen Exome Research Panel v2 (consisting of 5’biotin modified 415,115 oligonucleotide probes that span a 34 Mb target region of 19,433 genes) (Integrated DNA Technologies, Coralville, IA, USA), the exonic regions of all 19,433 human genes were obtained. The probes were normalized before pooling to ensure each probe represented in the panel at the correct concentration. After capturing, all captured regions were sequenced using a NovaSeq 6000 system (Illumina, San Diego, CA, USA). We obtained > 98.9% and > 99.2% of target sequences with ≥ 20× and ≥ 10× coverage, respectively. To convert and demultiplex base call sequence files to FASTQ files, bcl2fastq v2.20.0.422 (as NovaSeq files require bcl2fastq v2.19 or higher) (https://emea.support.illumina.com/downloads/bcl2fastq-conversion-software-v2-20.html) was utilized. After the sequencing, data was aligned to the GRCh37/hg19 human reference genome, variant calling, and annotation were performed using BWA-mem 0.7.17 (Burrows-Wheeler Aligner) (arXiv:1303.3997 [q-bio.GN]) to create BAM files. To generate VCF files, single nucleotide variants and minor insertions/deletions (indel) variant calling were performed on BAM data using GATK best practices (GATK, version 3.8; https://www.broadinstitute.org) [12, 13]. Utilizing depth-of-coverage (DOC) data, Conifer [14], and 3bCNV (https://3billion.io/resources) (Dated:16.06.2021), the copy number variant calling was performed. The AutoMap v1.2, which takes the VCF file (containing genotype and allelic depths for the reference and altered allele) as input, mapped the regions of homozygosity (ROH) from the VCF file [15]. AutoMap gave a text file (which contains detected ROHs) and a pdf file (which includes a graphical representation of ROHs) as an output. It took about 30 s to compute the exome data.

Variants were chosen based on the phenotype of each patient and the ACMG recommendations using the EVIDENCE tool (https://3billion.io/resources). This approach involves three essential steps: variant filtration, categorization, and similarity score for the patient’s phenotype. In the first step, the 3 billion genome database (https://3billion.io/main) and a genome aggregation database (gnomAD; http://gnomad.broadinstitute.org) were used for allele frequency estimation (Dated:17.06.2021). Gene variants with over 5% allele frequency were filtered out following ACMG recommendations. The Human Gene Mutation Database (HGMD) (https://www.hgmd.cf.ac.uk/ac/all.php) Professional 2022.1, Pakistan Genetic Mutation Database (PGMD) (https://www.pakmutation.kust.edu.pk), [11] the Database of Single Nucleotide Polymorphism (dbSNP) (https://www.ncbi.nlm.nih.gov/snp/), ClinVar (https://www.ncbi.nlm.nih.gov/clinvar), and VarSome [16] databases were used (Dated:18.10.2021) to assess the variants. Next, each variant related to the medical condition phenotype was evaluated by applying the ACMG standards [17]. Lastly, human phenotype ontology (HPO) (https://hpo.jax.org) provided a computational framework for organizing and displaying phenotypic data for further processing, and the resulting data was retrieved to assess the degree of similarity [18, 19] with each of the 7000 rare genetic diseases (https://omim.org/andhttps://www.orpha.net/consor/cgi-bin). As per the ACMG recommendations, there was a range of 0 to 10 in the similarity score between the phenotype of each patient and the disease-related symptoms resulting from priority variations. Medical geneticists and doctors then assess potential changes and associated conditions manually. We employed bidirectional Sanger sequencing to confirm the segregation of disease-causing variants within the families.

### Prediction and confirmation of structural analyses

Protein structures were retrieved for protein structural analysis from the Alpha Fold protein structure database, accessible online at https://alphafold.ebi.ac.uk [20]. To validate the 3D-modeled structure of the protein and to verify the phi and psi angles, the Ramachandran plot and the ERRAT (https://saves.mbi.ucla.edu/) were created using Procheck (https://saves.mbi.ucla.edu/) [21].

### Protein mutagenesis

Chimera (https://www.rbvi.ucsf.edu/chimera) and Biovia Discovery Studio (https://www.3ds.com/products-services/biovia/products/molecular-modeling simulation/biovia-discovery-studio/) were utilized to ascertain the 3D-structure of the mutant protein, which is significantly different from the wild-type protein. The mutant structure was derived from the wild-type protein. Biovia Discovery Studio examined the structural variations between wild-type and mutant proteins.

## Results

### Clinical features of family A

The affected individual (III-2) in family A displayed intellectual impairment symptoms such as hypotonia, lethargy, tremor, and developmental delays. A failure to thrive, a long face with a pointed chin, a high forehead, a flat philtrum groove, spasticity, an irregular stride, huge ears that were conspicuous, and nystagmus were all characteristics of his appearance. He displayed a terrible biting habit. He was unable to recognize different types of currencies and perform mathematical calculations. He also exhibited hostile behavior and did not engage in social engagement. The affected individual was observed to engage in actions such as self-biting and self-beating.

### Clinical features of family B

The clinical symptoms of III-2 in family B include a significant delay in the development of the child, difficulty speaking verbally, and unique facial traits. These included dolichocephaly, a flat face, thick eyebrows, widely spaced eyes, low-set ears, and a small nose. He also has anomalous hands and feet, such as malformed fingers or toes, joint deformities, or limb length variances. Deformities made it hard for him to grab, grip, walk, or balance. He also showed hyperphagia, or increased hunger and overeating. The patient has poor muscular tone, reducing strength and coordination. The person (IV-2) has a severe cognitive handicap that makes it difficult to learn, understand, and process information. He also had hyperactivity, impulsivity, and social difficulties. He was not able to walk or move and was not able to take care of himself. He was not able to perform the daily life activities and was handicapped. Key clinical findings in Family A and Family B have been mentioned in supplementary Table 1.

### Molecular findings

**Fig. 2:**
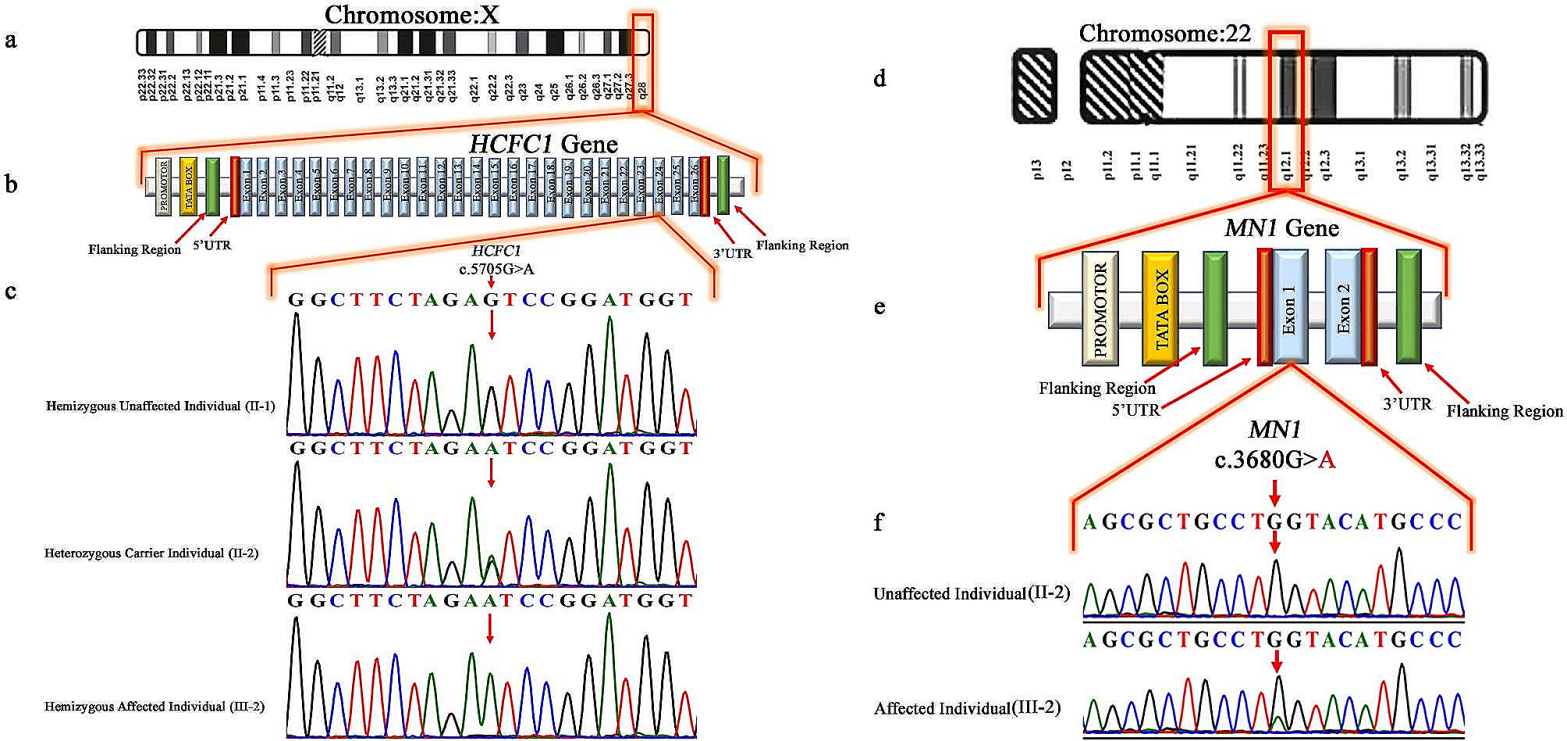
**(a)** Showing the location of HCFC1 gene (q28) on Chromosome X **(b)** Typical structure of HCFC1 gene comprising 26 exons and showing the location of the missense variant (c.5705G > A; p.Ser1902Asn) in exon-24 **(c)** Chromatograms of the Hemizygous unaffected individual (II-1) heterozygous carrier (II-2) and hemizygous affected individual (III-2) of Family A. **(d)** Showing the location of MN1 gene (q12.1) on Chromosome 22 **(e)** Typical structure of MN1 gene comprising 2 exons and showing the location of the nonsense variant (c.3680G > A; p.Trp1227Ter) in exon-1 **(f)** Chromatograms of the homozygous unaffected individual (II-2) and heterozygous affected individual (III-2) of Family B

Exome sequencing of Family A revealed a novel hemizygous missense variant (c.5705G > A; p.Ser1902Asn) in the *HCFC1* gene (NM_005334.3) on the X-chromosome. The segregation analysis of the variant revealed that the male individual (II-1 and III-1) were hemizygous wild-type (c.5705G), female individuals (II-2 and III-3) were heterozygous carriers (c.5705G/A), and the affected male individual (III-2) was hemizygous affected confirming the X-linked inheritance pattern of variant (Fig. 2a-c). Exome sequencing of Family B revealed a heterozygous nonsense variant (c.3680 G > A; p. Trp1227Ter) in exon-1 of the *MN1* gene (NM_032581.4). Sequencing analysis confirmed the segregation of this variant in the family, consistent with the autosomal dominant inheritance pattern. Unaffected individuals (II-2, III-1) of the family were homozygous wild-type (c.3680G/G), and the affected individual (III-2) was heterozygous affected (c.3680G/A) for this variant (Fig. 2d-f).

### Structural analysis of HCFC1 and MN1 protein

The wild-type HCFC1 and MN1 protein structures were obtained from the Alpha Fold protein structure database. These structures were assessed using ERRAT and PROCHECK. ERRAT analysis showed a High-quality score of 76.1218 and 95.8621 for HCFC1 and MN1 protein structures, demonstrating structure dependability. PROCHECK also showed that 81.0% of Ramachandran plot residues of MN1 protein were in the favorable region. These data confirm the protein structure’s good quality and conformation.

### Mutagenesis of HCFC1 protein and its comparison with wild-type

**Fig. 3:**
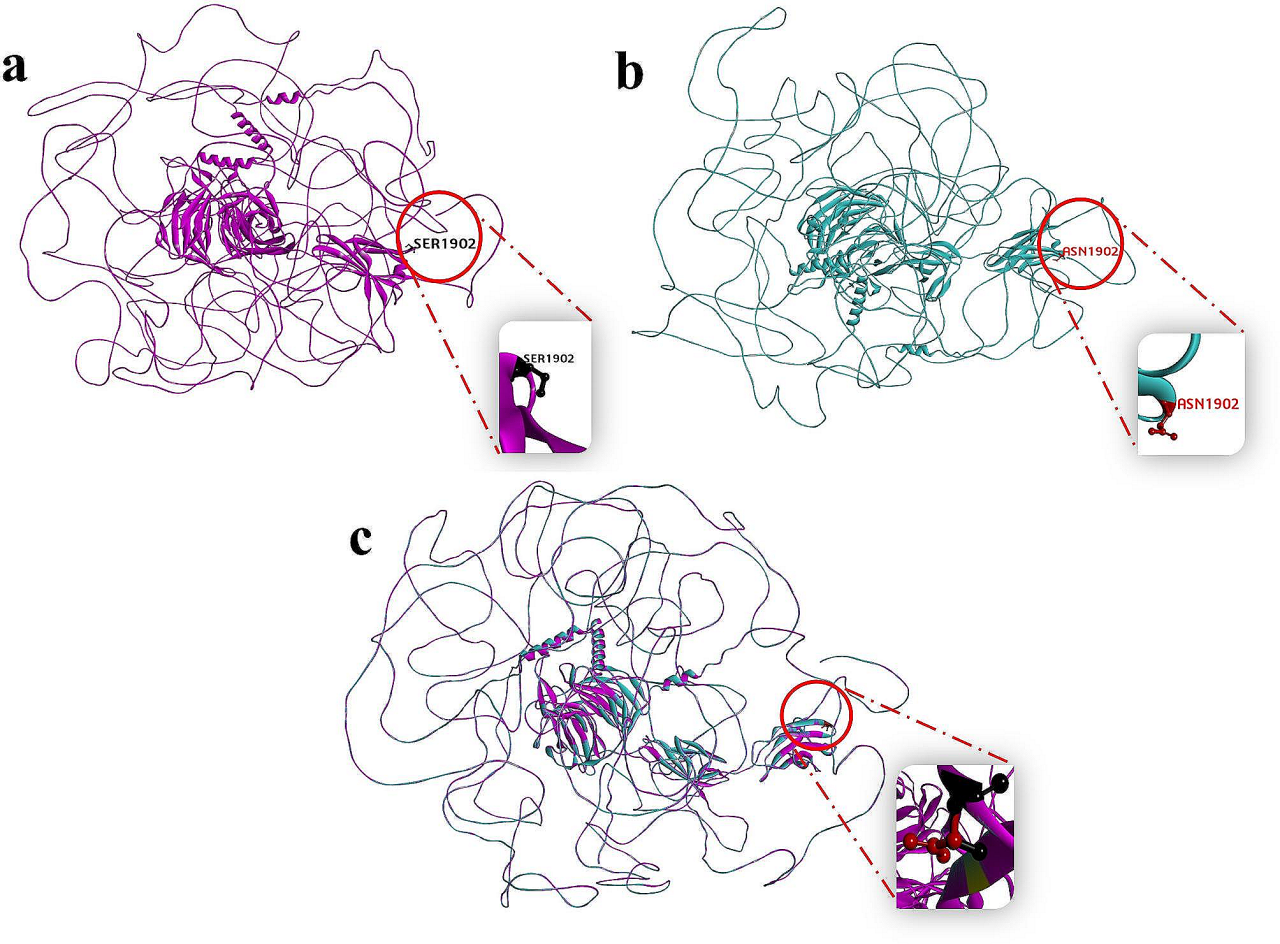
**(a)** Structure of wild type HCFC1 protein shown in pink and highlighting Ser1902 in black **(b)** Structure of mutant HCFC1 protein shown in green and highlighting Asn1902 in red **(c)** superimposed structure of wild type HCFC1 protein showing pink band and Ser1902 residue in black and mutant HCFC1 protein showing green band and Asn1902 residue in red color

The mutant HCFC1 protein structure was generated through mutagenesis using the chimera technique. The wild-type HCFC1 protein has a serine at position 1902. Mutagenesis in the wild-type HCFC1 protein was created using the Chimera tool by substituting asparagine in place of serine at position 1902 (Fig. 3). The wild-type and mutant HCFC1 protein structures were analyzed using Biovia Discovery Studio. Structures were superimposed and evaluated. The substitution of Ser1902Asn in HCFC1 confirmed abnormalities in the protein structure, affecting their wild-type conformation and function (Fig. 3).

### Homology modeling of MN1 and its comparison with wild-type

**Fig. 4:**
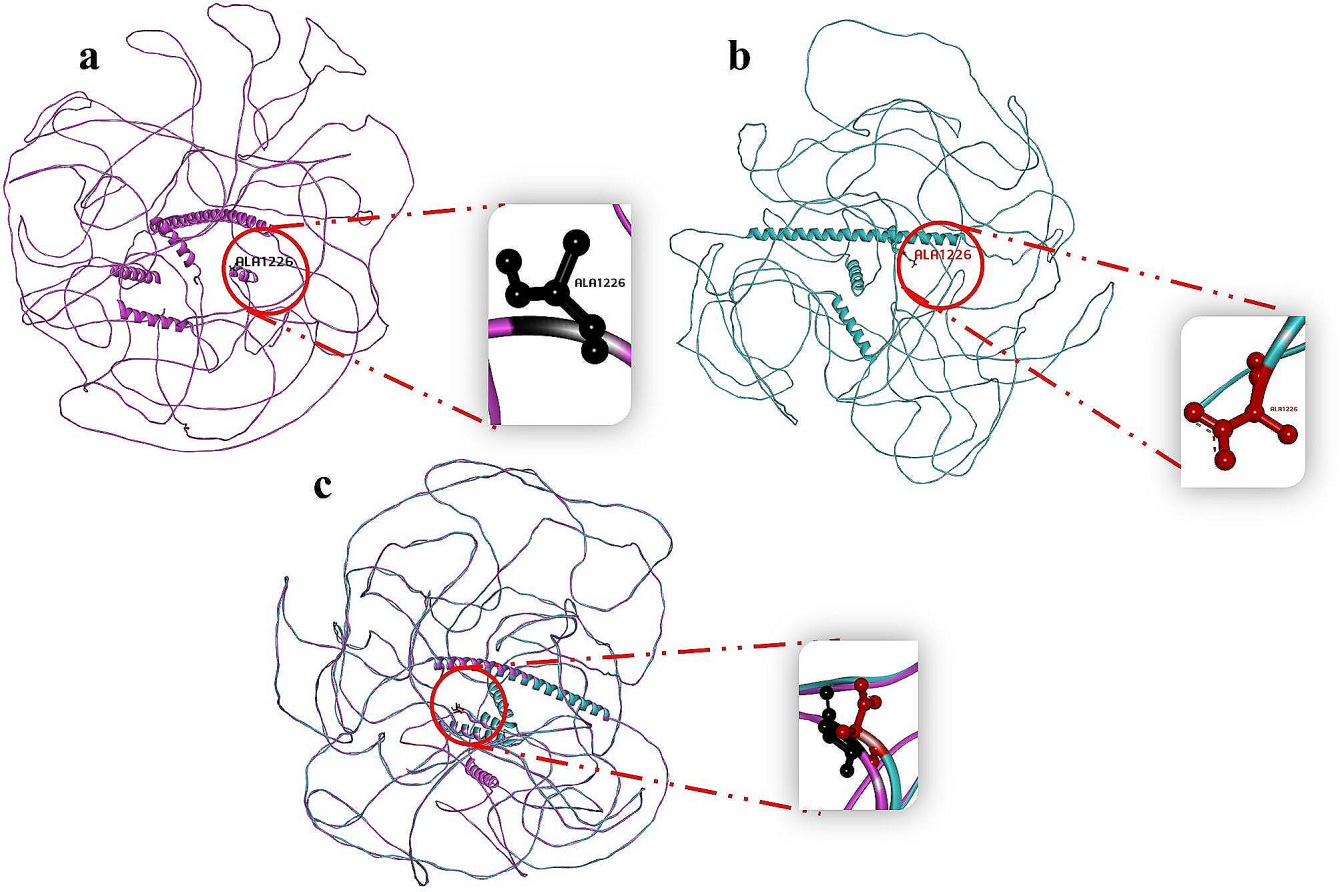
**(a)** Structure of wild type MN1 protein shown in pink and highlighting Ala1226 in black **(b)** Structure of mutant MN1 protein shown in green and highlighting Ala1226 in red **(c)** superimposed structure of wild type MN1 protein showing pink band and mutant MN1 protein showing green band indicating the premature truncation of the protein leading to ID phenotypes

The nonsense variant homology model was created using the SWISS Model. The wild-type MN1 protein has tryptophan at position 1227. A termination codon at position 1227 replaces the tryptophan amino acid in the mutant MN1 protein, terminating the sequence after the 1226 amino acid. Biovia-Discovery Studio evaluated wild-type and mutant MN1 protein structures. Superimposing and examining the structures showed that Trp1227Ter resulted in protein structural alterations. This premature protein truncation changed the protein’s structure and function (Fig. 4).

## Discussion

Intellectual disability (ID) affects a significant portion of the population, and the role of a substantial number of genetic factors in influencing normal brain functioning has been studied [22]. X-linked ID has been extensively studied, but it is not the primary cause of ID. Advances in research and technology have improved our understanding of the underlying causes, but the identification of pathogenic genetic variants is still ongoing [23]. Over 2,500 genes have been linked to ID, primarily caused by autosomal recessive ID [24]. Tools like homozygosity mapping and next-generation sequencing have been instrumental in these discoveries [25].

To date, 16 different mutations in *HCFC1* have been associated with various neurological abnormalities, including intellectual impairment, and the protein is involved in cellular growth and metabolism [3]. Previous studies have established a connection between *HCFC1* gene mutations and syndromic (cblX) and non-syndromic forms of intellectual impairment. Syndromic patients typically exhibit severe neurological abnormalities, including persistent epilepsy, facial dysmorphia, and intellectual impairment [3]. In the current study, we observed intellectual disability phenotypes with tremors and developmental delay. The affected individual presented self-beating and self-biting habits and failed to engage socially. The affected individual presented a long face with a pointed chin, a high forehead, a flat philtrum groove, spasticity, an irregular stride, huge ears and nystagmus phenotypes. Non-syndromic individuals with *HCFC1* mutations have also been documented, suggesting a potential role for *HCFC1* in brain development. The specific location of the mutation within the HCFC1 protein has been found to influence the severity of symptoms and the presence of an overall syndrome [4]. Previous studies have revealed a connection between *HCFC1* gene mutations and a complex disorder with a broad spectrum of symptoms affecting different organs and tissues [26].

Cobalamin deficiency, or cblX syndrome, is linked to mutations in the *HCFC1* gene’s kelch protein interaction domain. Other mutations in other domains of *HCFC1* do not lead to cobalamin deficiency, making these kelch domain mutations rare [27]. For instance, *HCFC1* promoter region mutations cause intellectual impairment but no cobalamin-related symptoms. However, mutations in the Kelch domain are always linked to intellectual impairment syndromes that have cobalamin-related symptoms. The p.Ser225Asn mutation once assumed to be a loss-of-function allele but now shown to modulate MMACHC production, but the effect this mutation on the protein function is still unclear [28].

Furthermore, the p.Ala477Asn variation in the fibronectin domain has been linked to intellectual impairment and facial dysmorphia, but not cobalamin deficiencies. Intellectual disability is commonly related to mutations in *HCFC1*’s essential region [28, 29]. The MMACHC promoter is mildly regulated by the p.Gly876Ser variation. Intellectual disability and aberrant brain development have been linked to additional C-terminal polymorphisms, such as p.Ala1756Val and p.Arg2016Trp, but not other traits [29]. *HCFC1* is a potential candidate gene for common partial epilepsy, demonstrating a unique mechanism of proteolysis dysfunction. Different functional roles are attributed to the various HCF-1 domains, with distinct clinical phenotypes linked to each, suggesting specific sub-molecular effects [4, 30]. In a previous study, seven hemizygous *HCFC1* variants were identified in 11 cases, a finding that was further validated in a cohort of 13 additional cases with six more hemizygous variants. All patients exhibited partial epilepsies without cobalamin disorders. The study highlighted that variants in the proteolysis domain were linked to mild and partial epilepsy, while those in the kelch domain were associated with cobalamin disorders characterized by severe and potentially fatal epileptic encephalopathy. Variants in the primary and acidic domains, on the other hand, were primarily related to intellectual disability [31].

Meningioma-1 (*MN1*) controls the proliferation, motility, differentiation, and function of osteoblasts in mammals as a transcription activator [32]. The brain deformity known as partial rhombencephalon synapsis is a highly uncommon condition defined by the midline fusion of the cerebellar hemispheres midline fusion and the cerebellar vermis’s loss by either a partial or whole extent. To date, 27 variants in the *MN1* gene have been reported to cause CEBALID syndrome [33]. At this point, there have been at least five patients who are documented to have variable craniofacial abnormalities (most of which involve cleft palate) and intellectual deficits [34–37]. These patients had deletions in the 22q12.1-q12.2 region that encompass the *MN1* gene. In a study that was conducted just a few years ago, a woman who was diagnosed with developmental delay, cleft palate, and an open anterior fontanelle was investigated. The researcher discovered that the woman had a deletion of 2.28 mega-bases that included the NF2 gene, to a loss of 1.61 mega-bases that included the *MN1* gene [38].

Two families from Pakistan, each with identification documentation, were selected and invited to participate in clinical and genetic investigations in the current study. In the family A, a novel hemizygous missense variant (c.5705G > A; p.Ser1902Asn) was identified in the *HCFC1* gene. This variant followed an X-linked inheritance pattern within the family, as confirmed by Sanger sequencing. The effect of the amino acid substitution on the protein structure was analyzed to gain further insights into the functional impact of the variant, which was achieved by comparing and superimposing the mutant and wild-type protein structures of HCFC1. The analysis revealed that the amino acid substitution resulted in altered interactions and a disrupted protein conformation. This alteration in the protein structure led to disorder within the protein. These findings highlight the potential significance of the identified variant in the *HCFC1* gene in the context of X-linked inheritance. The observed changes in protein structure and interactions suggest that this variant may contribute to developing specific phenotypic characteristics or disorders within affected individuals in family A. This hemizygous missense variant (c.5705G > A; p.Ser190Asn) of Family A was located in the fibronectin domain (FN3) of the *HCFC1* gene. Previous research has revealed two additional pathogenic variants (p.Ala477Asn & p.Arg2016Trp) located in the same FN3 domain of the *HCFC1* gene [3]. The pathogenic variant p.Ala477Asn was linked to intellectual disability and facial dysmorphia but not cobalamin defects [27]. On the other hand, the pathogenic variant p.Arg2016Trp was associated with intellectual disability and abnormal brain development without other observed phenotypes [3].

In Family B, an individual presented with a range of symptoms, including intellectual disability, severe developmental delay, poor language skills, dysmorphic facial features, skeletal deformities, hyperphagia, hyperactivity, and severe aggressive behavior. WES identified a novel heterozygous nonsense variant (c.3680G > A; p.Trp1227Ter) in the *MN1* gene after the filtration. This variant followed an autosomal dominant inheritance pattern within the family, meaning that affected individuals carried one copy of the variant (c.3680G/A), while unaffected individuals had two copies of the wild-type variant (c.3680G/G). Further analysis was conducted to understand the functional consequences of the identified variant. Structural analysis revealed significant conformational changes in the protein due to the premature stop codon caused by the variant. Additionally, the variant led to a reduction in the length of the protein. These structural changes and protein truncation are believed to contribute to the observed intellectual disability in the affected individual. The disruption of the *MN1* gene’s normal function likely plays a role in the manifestation of the various symptoms, including developmental delay, language difficulties, dysmorphic facial features, skeletal deformities, hyperphagia, hyperactivity, and severe aggressive behavior. This finding underscores the importance of understanding the molecular mechanisms underlying intellectual disability and highlights the role of the *MN1* gene in normal cognitive development.

## Conclusions

This study investigated the genetics of intellectual disability in two families and identified a novel genetic variant related to ID, expanding the genetic spectrum and variability of intellectual disability and improving our understanding of the condition. The use of next-generation sequencing has been beneficial in identifying genetic variants in families with hereditary disorders.

### Electronic supplementary material

Below is the link to the electronic supplementary material.

Supplementary Material 1

## Data Availability

We have submitted the variants data in ClinVar repository (https://submit.ncbi.nlm.nih.gov/clinvar/) under the accession numbers SCV004801669 and SCV004708216.
