# Supplementary material for: Variants in HCFC1 and MN1 genes causing intellectual disability in two Pakistani families
Source: BMC Med Genomics. 2024 Jul 2;17:176. doi: 10.1186/s12920-024-01943-2 (PMC11221130; doi:10.1186/s12920-024-01943-2)
Supplement: Supplementary file 1 — Supplementary Material 1 [file 12920_2024_1943_MOESM1_ESM.docx]

**Supplementary Table 1:** Summary of molecular genetics and key clinical findings in Family A and Family B.

| **Molecular genetics summary** | | |
| --- | --- | --- |
|  | **Family A** | **Family B** |
| Causative gene | ***HCFC1*** | ***MN1*** |
| Genomic position (GRCh37) | 153215993G>A | 28192852G>A |
| c. position | 5705G>A | 3680G>A |
| p. position | Ser1902Asn | Trp1227Ter |
| Variant Novelty | **Novel Variant** | **-** |
| CADD_Phred | 22.9 | 32 |
| MutationTaster | Disease Causing | Disease Causing |
| Bravo Topmed | Not Found | Not Found |
| gnomAD v.2.1.1 | Not Found | Not Found |
| Varsome (PhyloP100) | 4.96 | 7.329 |
| **Clinical Summary** | | |
| **Biometric Data** | | |
| Patient ID | III-2 | III-2 |
| Age | - | 25 years |
| Sex | Male | Male |
| **Onset of Disease** | | |
| Congenital or not | Yes | Yes |
| Disease onset age | By Birth | By birth |
| Family disease history | No | No |
| Attending any specialized educational institute | No | No |
| **Environmental Causes** | | |
| Any medical Complication (pre-natal & post-natal) | No | No |
| Drug usage by mother | No | No |
| Childhood disease | No | No |
| Head injury or trauma | No | No |
| psychological trauma | No | No |
| Depression | No | No |
| **Behavioral** | | |
| Excessively energetic | Yes | Yes |
| Drowsy | No | No |
| Depressed | Yes | No |
| Joyful | No | Yes |
| **Verbal Communication** | | |
| Speech development | Poor | No |
| Speech (single word or few words) | Yes | No |
| **Conceptual analysis** | | |
| Currency recognition and counting | No | No |
| Self-care | Yes | No |
| Parental recognition | Yes | Yes |
| Residence recognition | Yes | No |
| Repeat behavior | Yes | No |
| Cognitive learning ability | Poor | No |
| **Growth Condition** | | |
| Normal Physique | Yes | No |
| Weak physique | No | Yes |
| Truncal obesity present or not | No | No |
| **Neurological Examination** | | |
| Intellectaul Disability | Moderate | Severe |
| Epilepsy | No | Yes |
| Facial expression | Dull | Active |
| Gaitk | Abnormal | No |
| Muscle tone (normal or weak) | Hypotonia | Weak |
| Ambulation | Yes | Yes |
| Muscular atrophy | No | No |
| High arch feet (Pes Cavus) | No | No |
| Limb’s dystonia | No | Yes |
| Dysarthric speech | No | Yes |
| Abnormal spine curvature (Kyphosis) | No | Yes |
| **Adaptive Skills** | | |
| Self-Feeding | Yes | No |
| Toilet training | Poor | No |
| Dressing/undressing training | Yes | No |
| Reading/Writing | No | No |
| Self-care | Yes | No |
| Pay attention/concentration | No | No |
| **Facial Features** | | |
| Philtrum | Flat | Flat |
| Forehead protruding or not | Yes | No |
| Neck webbing | No | Yes |
| Skeletal abnormalities | No | Yes |
| Microcephaly | No | No |
| Macrocephaly | No | No |
| Ptosis | No | Yes |
| High nasal bridge | No | Yes |
| Prominent Nose | No | No |
| Long Face | No | Yes |
| Spasticity | No | No |
| Hypotonia | Yes | No |
| Polydactyly condition | No | No |
| Syndactyly condition | No | No |
| Bracydactyly condition | No | Yes |
| hypertelorism | No | No |
| camptodactyly | No | No |
| Deep tendon reflexes | Yes | Yes |
| Short stature | No | No |
| Dental abnormalities | No | No |
| **Skin Texture** | | |
| Freckling of skin | Yes | No |
| Skin morphology | Dry | Normal |
| Nail shape and development | Normal | Normal |
| Development of hairs | Thick hair | Normal |
| Allergic skin | No | No |
| **Miscellaneous** | | |
| Impairment in hearing | No | No |
| Allergic response to any food | No | No |
| Early diagnosis, treatment and efficiency | No | No |
